# An Unusual Case of Rhizobium radiobacter in Bronchoalveolar Lavage

**DOI:** 10.7759/cureus.92808

**Published:** 2025-09-20

**Authors:** Ezaz Yousaf, Rizwan Pervaiz, Muhammad A Rana, Anum Mehboob, Sitara Raza

**Affiliations:** 1 Internal and Critical Care Medicine, Bahria International Hospital, Lahore, PAK; 2 Internal, Respiratory, and Critical Care Medicine, Bahria International Hospital, Amna Inayat Medical College, Lahore, PAK; 3 Critical Care Medicine, Bahria International Hospital, Lahore, PAK; 4 Intensive Care Unit, Bahria International Hospital, Lahore, PAK

**Keywords:** bronchoalveolar lavage, catheter associated, end-stage renal disease (esrd), fungal culture, sars-cov-2

## Abstract

*Rhizobium radiobacter* is a gram-negative bacterium that causes tumors in plants. In humans, it rarely causes infections. In most of the reported cases, the patient had long-standing indwelling devices or immunocompromised states. We present a case of a 49-year-old male with end-stage renal disease (ESRD) who is on twice-weekly maintenance hemodialysis, presenting with shortness of breath. With the initial diagnosis of community-acquired pneumonia and high-resolution computed tomography findings, bronchoscopy was performed, and bronchial washing was sent for pneumonia panel and cultures. The pneumonia panel was positive for severe acute respiratory syndrome coronavirus 2 (SARS-CoV-2), and the culture yielded growth of *Rhizobium radiobacter* and *Aspergillus*. Bronchial washing was also positive for COVID-19 infection. Antibiotics and antifungals were started, and the patient's inflammatory markers began to improve. To the best of our knowledge, this is the first case reported in Pakistan and depicts the importance of proper and timely evaluation of infections in immunocompromised patients.

## Introduction

Most of the cases of community-acquired pneumonia worldwide are caused by bacteria, viruses, and fungi [1]. Common bacterial pathogens are gram-positive agents (*Streptococcus pneumoniae, Staphylococcus aureus, group A streptococci*, and other streptococci), gram-negative agents (*Haemophilus influenzae, Moraxella catarrhalis, and Enterobacteriaceae*), and atypical agents (*Mycoplasma, *Legionella, *Chlamydia pneumoniae, and Chlamydia psittaci*) [2]. Among viruses, severe acute respiratory syndrome coronavirus 2 (SARS-CoV-2), influenza, rhinovirus, respiratory syncytial virus, parainfluenza, and human metapneumovirus are increasingly detected as pathogens based on molecular detection methods. Worldwide, *Streptococcus pneumoniae* and *Haemophilus influenzae* are still the leading causes of acute bacterial pneumonia. The most recent United States population-based surveillance showed that *S. pneumoniae*, the influenza virus, and the human rhinovirus were the most common causative pathogens [3].

*Rhizobium radiobacter* (formerly *Agrobacterium tumefaciens*) is a gram-negative, non-spore-forming, motile, and aerobic rod that usually causes tumors in plants. Its natural habitat is soil, along with other non-fermenting bacteria. Human disease caused by the genus *Rhizobium *is an uncommon phenomenon. It is transmitted to humans by contaminated medical devices like intravenous catheters or direct exposure of a traumatic wound to soil. There are five known species of bacteria in the genus *Rhizobium*. Interestingly, *R. radiobacter*, despite its low virulence, is the only species of the genus *Rhizobium* involved in the development of diseases in humans [4].

## Case presentation

We report a 47-year-old male with end-stage renal disease (ESRD) on maintenance hemodialysis, who presented to the emergency room with a three-day history of fever and progressive shortness of breath. Initial vitals showed a fever of 102°F, a heart rate of 133 beats/minute, a blood pressure of 155/88 mmHg, and an SpO_2_ of 78% at room air, which improved to 92% with 10 liters of oxygen via face mask. On physical examination, we had a young man with diaphoresis, in visible distress, with a double-lumen dialysis catheter in the right internal jugular vein and bilateral coarse crackles on chest auscultation.

**Figure 1:**
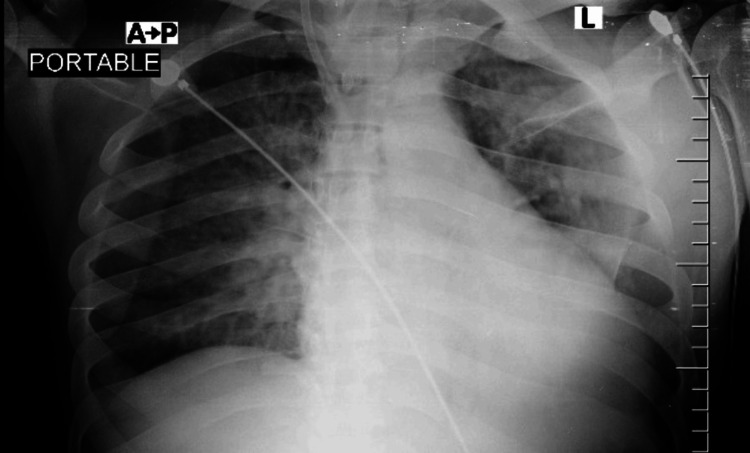
Initial chest X-ray reveals ground-glass changes.

A diagnostic workup was initiated, including complete blood counts, venous blood gases, a metabolic panel, and a viral respiratory panel. Two sets of blood cultures from the double-lumen catheter and the peripheral line were taken and sent for culture and sensitivity, and the catheter was removed. The patient's initial venous blood gas report was unremarkable. A complete blood count revealed a normal white blood cell count, anemia, and an elevated platelet count (Table 1). Other notable laboratory studies included elevated lactic acid, C-reactive protein, and procalcitonin levels. The viral respiratory panel from the nasopharyngeal swab sample was negative. Chest X-ray (Figure 1) showed bilateral interstitial opacities.

**Table 1:** Initial laboratory investigations CRP = C-reactive protein, Hb = hemoglobin, WBC = white cell count, PaCO_2 _= partial pressure of carbon dioxide, PaO_2 _= partial pressure of oxygen

Parameter	Patient Value	Reference Value
Sodium	139 mmol/L	135–145
Potassium	4.5 mmol/L	3.5–5.1
Creatinine	6.72 mg/dL	0.7–1.3
CRP	150.0 mg/L	<5
Hb	7.92 g/dL	4–11
WBC	8.95 × 10⁹/L	13.5–16.5
Platelets	512 × 10⁹/L	150–450
Procalcitonin (PCT)	7.25 ng/mL	<0.07
Lactate	3.5 mmol/L	0.5–2.2
pH	7.46	7.35–7.45
PaCO_2_	37 mmHg	35–45
PaO_2_	33 mmHg	80–100
Bicarb	28 mmHg	22–26

**Figure 2:**
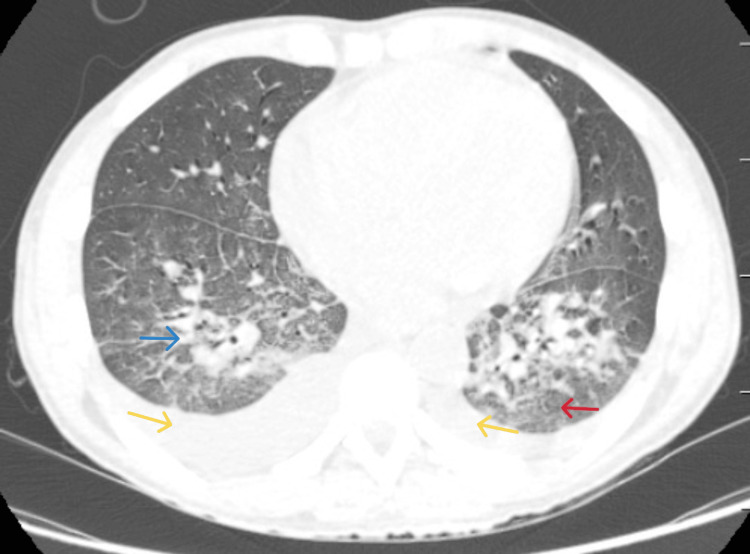
High-resolution computed tomography at admission showing bilateral scattered nodular infiltrates (blue arrow), ground-glass changes (red arrow), and bilateral pleural effusions (yellow arrows).

With an APACHE II score of 21, the patient was admitted to the intensive care unit (ICU) with suspicion of acute community-acquired pneumonia. High-resolution computed tomography of the thorax (Figure 2) at admission showed bilateral mediastinal lymphadenopathy and bilateral subsegmental atelectasis and ground-glass opacities. Bronchoscopy with bronchoalveolar lavage (BAL) was performed the same day, and bronchial washing was sent for a pneumonia panel and bacterial and fungal cultures. Antibiotics were started empirically according to the hospital antibiogram. The pneumonia panel was positive for SARS-CoV-2, and the culture grew *Rhizobium radiobacter* (sensitive to amikacin, ceftriaxone, ciprofloxacin, imipenem, gentamicin, meropenem, piperacillin/tazobactam, and cotrimoxazole) and *Aspergillus* species. Blood cultures were negative for any bacterial growth.

Voriconazole was started for *Aspergillus*,* *meropenem for *R. radiobacter*, and vancomycin for gram-positive coverage. The patient began to show improvement after transitioning to a more specific antimicrobial regimen. His fever settled down, his oxygen requirement decreased, and inflammatory markers started to improve. Three days after initiation of antibiotics, the patient was stepped down from the ICU. The patient made an uneventful recovery and was ultimately discharged home.

## Discussion

Post-COVID pneumonia is increasingly recognized as a complex clinical entity often involving secondary bacterial and fungal infections due to impaired host immunity. *R. radiobacter* is an unusual cause of pneumonia, with most reported cases occurring in patients with central venous catheters, malignancy, or other causes of immunosuppression [5].

The earliest report of *R. radiobacter* causing endocarditis following aortic valve replacement dates back to 1980 [6]. Since the early 2000s, increasing numbers of infections have been reported, including endocarditis post-heart valve replacement [7], bacteremia in cancer patients [8], neonatal sepsis [9], peritonitis [10], endophthalmitis [11], UTIs in dialysis patients, nosocomial pneumonia [12], and brain abscesses [13]. In addition, cases of these microorganisms in the development of UTIs (in patients requiring hemodialysis), brain abscesses, and nosocomial pneumonia, among others, have been reported [14]. The placement and presence of plastic devices in the human body is a major factor in the development of infections caused by this *R. rhizobacter*. This can be attributed to the high adhesion properties of rhizobia to plastic/silicone surfaces, which are due to the production of extracellular mucus [15].

In our patient, risk factors included ESRD, requiring long-term catheterization, and concurrent COVID-19. The source of *R. radiobacter* infection remains uncertain, but hematogenous seeding from the catheter or inhalational exposure cannot be excluded.

Currently, the list of antibiotics for susceptibility testing on *R. radiobacter* is undefined. The European Committee on Antimicrobial Susceptibility Testing (EUCAST) guidelines, while authoritative, did not include this organism in their 2025 update, creating uncertainty around appropriate testing standards [16]. This gap requires further discussion within the microbiological and infectious disease community.

There is limited literature on treatment protocols for *R. radiobacter*;* t*reatment is typically guided by susceptibility testing, and there is limited data on antimicrobial resistance. In our case, the organism was susceptible to the chosen targeted therapy, and the clinical response was favorable.

## Conclusions

To our knowledge, this is the first reported case of *R. radiobacter* pneumonia in Pakistan. Although still limited to a few sporadic case reports, the increasing recognition of this infection highlights its rising clinical relevance in immunocompromised patients, especially when we are living in the post-COVID era. In a world already struggling with multidrug-resistant organisms, the emergence of such unusual infections serves as a warning sign that our index of suspicion must continually stay wide. Early microbial identification, prompt initiation of targeted therapy, and meticulous infection control measures remain pivotal to improve outcomes and limit their potential spread. Publishing such cases not only contributes to the existing literature but also provides a foundation for improving our diagnostic vigilance and shaping the future therapeutic strategies against emerging opportunistic pathogens in times when there are limited new antibiotics in the pipeline.

The purpose of writing the case report is to pass on this message to clinicians to remain vigilant for rare opportunistic infections like the one we have come across, as early recognition and effective infection control are our strongest defenses in an era of rising antimicrobial resistance.
